# Targeting vaginal dysbiosis: prospects for the application of live biotherapeutics products

**DOI:** 10.3389/fmicb.2026.1749581

**Published:** 2026-01-28

**Authors:** Qiongqiong Zhang, Xiaoxiao Zhao, Zhangran Chen, Rui Chen, Xiong Lin, Lei Zhang, Kangning Li, Min Wang, Yanmin Liu, Huan Zhou, Qinping Liao

**Affiliations:** 1 Department of Obstetrics and Gynecology, Beijing Tsinghua Changgung Hospital, School of Clinical Medicine, Tsinghua Medicine, Tsinghua University, Beijing, China; 2 Shenzhen Wedge Microbiology Research Co., Ltd, Shenzhen, China

**Keywords:** *Lactobacillus*, live biotherapeutics products, microbiota-based therapy, vaginal dysbiosis, vaginal microbiota

## Abstract

As a pivotal defense system within the female lower genital tract, the healthy vaginal microecosystem, dominated by *Lactobacillus*, safeguards against pathogenic microorganisms and maintains overall reproductive health through producing antimicrobial substances and sustaining an acidic environment. However, this intricate ecosystem is susceptible to a variety of adverse factors that trigger vaginal microbiota (VMB) dysbiosis, which further precipitate vaginal infections and gynecological disorders. Based on rigorous clinical evidence, this review systematically summarizes current mechanistic understanding of *Lactobacillus*-mediated VMB homeostasis. It evaluates the therapeutic potential of probiotics in both pharmaceutical and dietary supplement forms, and discusses the clinical necessity and existing challenges in developing live biotherapeutic products (LBPs) targeting the vaginal microecology. By integrating perspectives from both basic research and translational medicine, this work provides a theoretical foundation for developing targeted microbiota modulation strategies, thereby advancing precision medicine approaches for the management of vaginal dysbiosis.

## 1 Introduction

The vaginal microbiota (VMB) has emerged as a central research focus not only in contemporary microbiology and gynecology, but also in obstetrics (France et al., 2022; Hong et al., 2022; Li and Wu, 2024; Mandelbrot et al., 2025). Substantial evidence indicates that a *Lactobacillus*-dominant VMB is strongly associated with optimal reproductive health, whereas dysbiosis is closely linked to adverse outcomes, including vaginitis (Leclair and Stenson, 2022), endometritis (Wang et al., 2021), pelvic inflammatory diseases (Haggerty and Ness, 2006), secondary infertility (Zhao et al., 2020), adverse pregnancy outcomes (Grewal et al., 2022), and increasing susceptibility to human papillomavirus (HPV) and human immunodeficiency virus (HIV) infections (McClelland et al., 2018; Mei et al., 2022). Although antibiotic therapy remains the standard clinical intervention for bacterial vaginosis (BV), its limitations—such as high recurrence rates (40%–60%) and the emergence of antimicrobial resistance (Muzny and Sobel, 2023; Mayr et al., 2024) —have driven researchers to explore alternative strategies, including *Lactobacillus*-based live biotherapeutic products (LBPs) and other microbiota-modulating approaches. Research has revealed that *Lactobacillus* exerts its protective effects through multifaceted mechanisms. Beyond maintaining a low vaginal pH, these bacteria produce antimicrobial compounds such as like lactic acid (O’Hanlon et al., 2019), bacteriocins (Zhang et al., 2023), and potentially hydrogen peroxide (H_2_O_2_) (Miko and Barakonyi, 2023), which inhibit pathogen proliferation via direct antimicrobial activity, competitive adhesion, and immune modulation (Doerflinger et al., 2014; Pino et al., 2024). Notably, multicenter studies have demonstrated significant racial and geographic variations in VMB composition, likely influenced by host genetics, environmental exposures, and behavioral factors. Additionally, dynamic variables such as menstrual cycle fluctuations, sexual activity, and antibiotic use further complicate VMB stability, underscoring the need for personalized microbiota-targeted interventions (Ferrer et al., 2017; France et al., 2022; Houttu et al., 2022).

While LBPs show promise in treating and preventing BV and vulvovaginal candidiasis (VVC), key challenges remain, including clarifying their mechanisms of action, ensuring long-term safety, and optimizing clinical application protocols (Liu et al., 2023). With the development of multi-omics technology, our understanding of vaginal health and disease has been significantly improved, offering new avenues for the microbiota-targeted treatment of gynecological diseases (Huang et al., 2024).

In the current field of VMB research, clinical practice still lacks precise and effective intervention strategies. Against this backdrop, we have prepared this comprehensive review. This review not only provides a thorough summary of the latest research findings on *Lactobacillus*-mediated VMB homeostasis but also offers an in-depth examination of the current status, existing problems, and challenges faced by LBPs in clinical applications. By deeply integrating mechanistic insights from fundamental research with robust evidence from clinical practice, we aim to provide clear guidance for the development of next-generation microbiota-targeted therapies, ultimately advancing personalized management of vaginal dysbiosis to new heights.

## 2 The vaginal microbiota

### 2.1 History of vaginal microbiota classification

The study of VMB has undergone a series of conceptual shifts, each marked by technological advances that deepened our understanding of host–microbe interactions. Initial cultivation-based approaches established a dichotomous framework contrasting a *Lactobacillus*-dominated “healthy” state with BV, characterized by anaerobic overgrowth (Fredricks et al., 2005). This view, however, was limited by the inherent constraints of cultural methods.

The introduction of high-throughput 16S rRNA gene sequencing represented a turning point. Ravel et al.’s landmark 2011 study applied unsupervised clustering to data from healthy women, revealing substantial interindividual heterogeneity and leading to the Community State Types (CSTs) classification. This system groups VMB into five major types (CST-I to CST-V), reframing the field from a binary perspective to a spectrum-based model and providing a common vocabulary for comparative research (Ravel et al., 2011).

Subsequent progress in metagenomics and metatranscriptomics—supported by dedicated databases—enabled finer-grained profiling. The VIRGO database offered the first systematic integration of reference genomes and functional annotations for vaginal microbes (Ma et al., 2020). Its successor, VMGC, integrated large-scale datasets to significantly broaden taxonomic coverage and improve resolution in strain-level typing and functional annotation (Huang et al., 2024).

More recently, large-scale population studies have enabled even finer subclassification into vagitypes. For example, one analysis distinguished 13 such types, subdividing the heterogeneous CST-IV category into subtypes defined by anaerobes such as *Gardnerella* and *Prevotella*, and uncovering health links tied to subtle structural variations. Illustratively, vagitypes dominated by *L. iners* and *L. jensenii* were associated with higher live birth rates—a finding that challenges the notion of *L. iners* as merely weakly protective, and underscores that health outcomes are shaped by community structure rather than individual species (Qin et al., 2025).

### 2.2 Influencing factors

**FIGURE 1:**
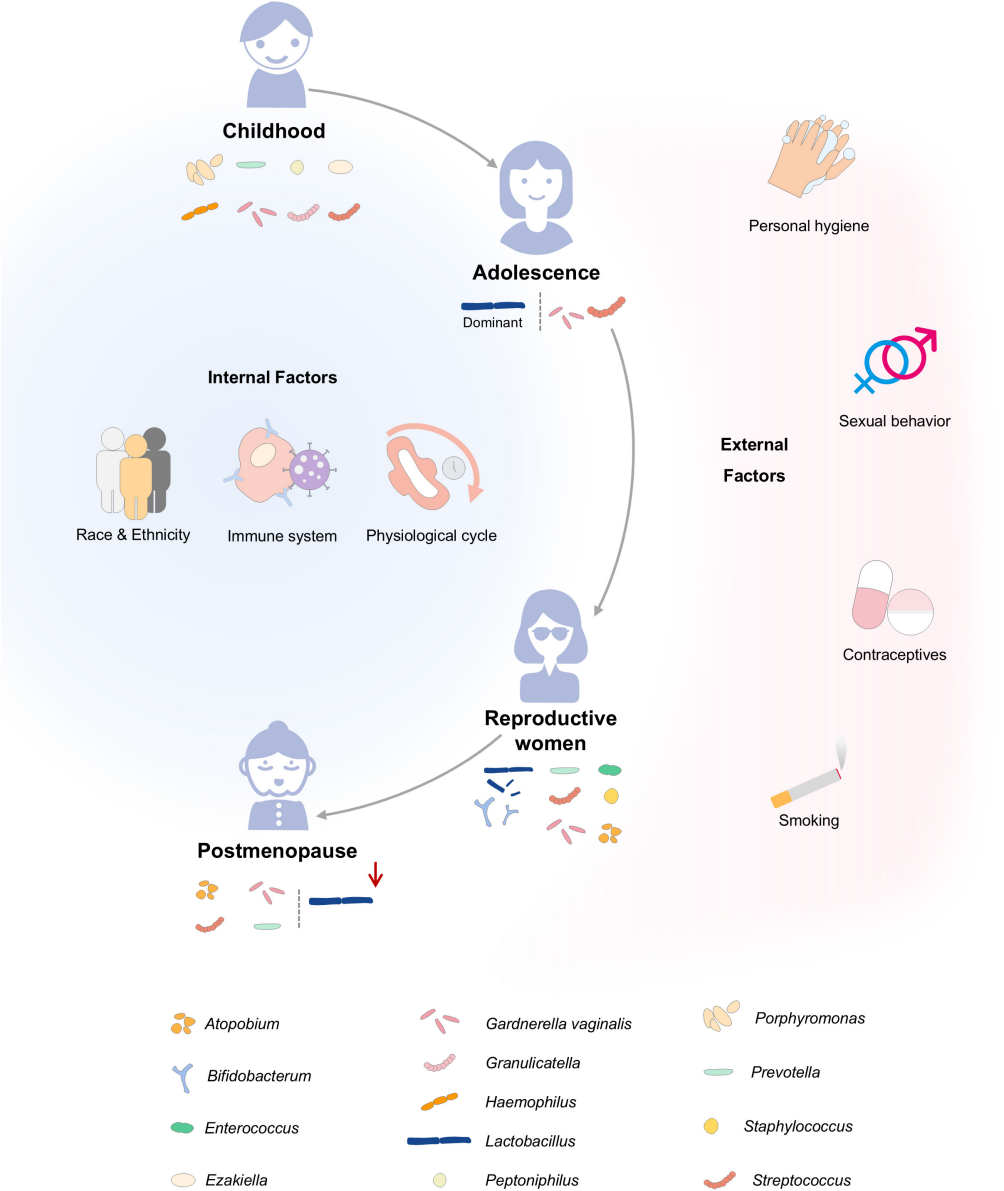
Dynamic changes and influencing factors of vaginal microbiota (VMB) across the female lifespan. Arranged horizontally from left to right, the timeline depicts four main stages: childhood, adolescence, childbearing years, and postmenopause. Internal factors (left panel) include genetic, immunological, and physiological influences, while external factors (right panel) encompass behavioral and environmental exposures. Dominant microbial taxa are color-coded and listed beneath each life stage, showing the dynamic shifts in microbial communities.

The VMB’s structure and function are dynamically regulated by intrinsic and extrinsic factors, which collectively influence the succession and health functions of the VMB throughout a woman’s lifetime (Figure 1; Lehtoranta et al., 2022; Qi et al., 2025).

During childhood and pre-pubertal stages, the vaginal pH remains neutral or alkaline, with the microbial community primarily composed of aerobic and strict anaerobic bacteria, where *Lactobacillus* are only occasionally detected (Hu et al., 2021; Xiaoming et al., 2021). As ovarian maturation occurs and estrogen levels surge during puberty, the vaginal microbiome undergoes significant remodeling, transitioning to a *Lactobacillus*-dominated ecosystem (Yamamoto et al., 2009). In postmenopausal women, declining estrogen levels reduce glycogen availability in vaginal epithelial cells. This leads to a gradual depletion of *Lactobacillus* populations and their subsequent replacement by CST-IV type bacteria, resulting in an elevated vaginal pH and increased susceptibility to infections (Shardell et al., 2021). External factors such as sexual practices, hygiene routines, antibiotic use, and contraceptive choices can directly disrupt the VMB balance (Auriemma et al., 2021; Holdcroft et al., 2023; Miller et al., 2024). Pathological conditions, such as BV, VVC, aerobic vaginitis (AV), cytolytic vaginosis (Rouanet et al.), trichomonas vaginitis (TV), atrophic vaginitis, and mixed vaginitis altering its composition (Brotman et al., 2018; Kim et al., 2020; Yang et al., 2020a; Qi et al., 2021; Gaziano et al., 2023; Muzny et al., 2023). For example, BV is characterized by the displacement of *Lactobacillus* and overgrowth of facultative anaerobes such as *G. vaginalis*, and anaerobic bacteria including *Prevotella*, *Fannyhessea vaginae*, *Sneathia*, and *Megasphaera* (Muzny et al., 2023).

### 2.3 Protective mechanisms of *Lactobacillus*

**FIGURE 2:**
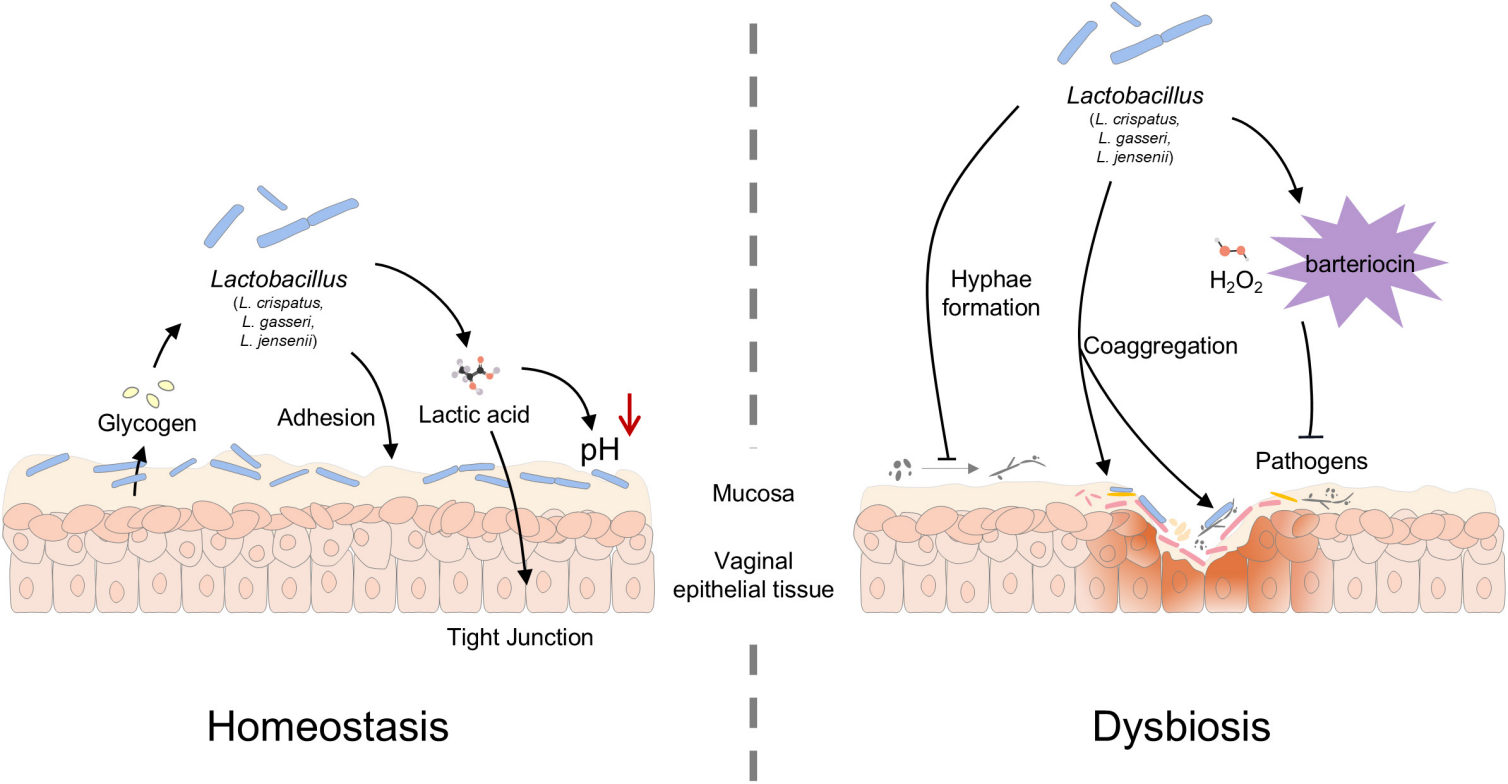
Role of *Lactobacillus* in maintaining a balanced vaginal microbiota (VMB). *Lactobacillus* adheres to vaginal epithelial cells in a homeostatic vaginal environment and decomposes the released glycogen to produce lactic acid, lowering the vaginal pH and tight the vaginal epithelium. *Lactobacillus* also inhibits the growth and hyphae formation of pathogenic bacteria through bacteriocin, H_2_O_2_, and coaggregation.

*Lactobacillus* including *L. crispatus, L. gasseri*, and *L. jensenii* are crucial for safeguarding the vaginal epithelium against pathogenic infections, and their protective role can be attributed to several key mechanisms (Figure 2). Firstly, *Lactobacillus* firmly adhere to the vaginal epithelium and create a biological barrier including surface proteins and polysaccharides. This barrier not only stabilizes their colonization but also acts as a shield on the epithelial surface, effectively preventing the attachment of pathogenic microorganisms and inhibiting the development of pathogenic biofilms (Abramov et al., 2023). Secondly, *Lactobacillus* contributes to the maintenance of the vaginal acidic milieu by metabolizing glycogen from epithelial cells and fermenting it into lactic acid. This process lowers the vaginal pH, thereby creating a hostile environment for acid-sensitive microbes (O’Hanlon et al., 2019). Thirdly, *Lactobacillus* produces bacteriocins, which are antimicrobial compounds that help defend the genital tract against infections by eliminating pathogenic bacteria (Zhang et al., 2023).

Furthermore, a wealth of epidemiological studies supports the protective role of H_2_O_2_ derived from lactobacilli. Its mechanism of action likely does not rely on substantial accumulation in vaginal fluid, but rather exerts its effects within the local microenvironment where lactobacilli are in close contact with adjacent microorganisms. At these sites of interaction, the oxidative activity of H_2_O_2_ can inhibit the growth of various indigenous and exogenous bacteria. Notably, the production of H_2_O_2_ is often regarded as a biomarker of healthy, competitively superior *Lactobacillus* strains, associated with stronger colonization and ecological regulatory capabilities. However, its actual *in vivo* effects are constrained by the vaginal microaerophilic environment—which limits its generation—and the neutralizing actions of bodily fluids (O’Hanlon et al., 2010; Miko and Barakonyi, 2023). In contrast, *L. iners* offers less protection against the colonization of potentially harmful pathogens in the vaginal environment (France et al., 2016). The reduced protective efficacy of *L. iners* is primarily due to its inability to synthesize D-lactic acid. Studies demonstrate that D-lactic acid possesses significantly stronger antimicrobial activity against exogenous pathogens compared to its L-lactic acid (Witkin et al., 2013).

## 3 Vaginal microbiome-based intervention methods

**FIGURE 3:**
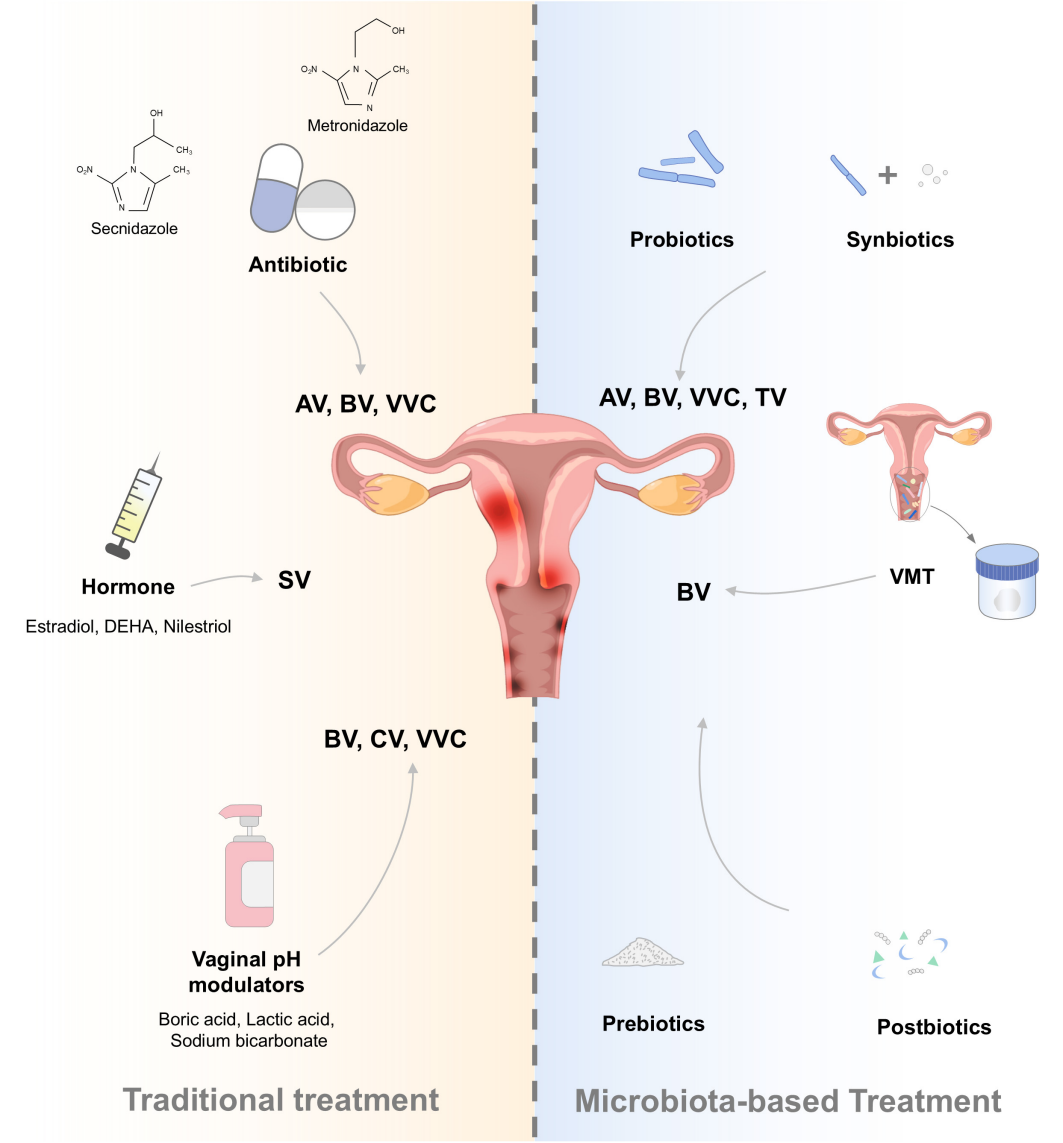
Traditional and microbiota-based treatments for vaginal diseases. AV, aerobic vaginitis; BV, bacterial vaginosis; CV, cytolytic vaginosis; TV, trichomonas vaginitis; VVC, vulvovaginal candidiasis; SV, senile vaginitis; DEHA, dehydroepiandrosterone. The vagina diagram was created using Figdraw (www.figdraw.com).

Vaginitis presents a dilemma for both patients and healthcare practitioners. Advances in VMB research and therapeutic development have resulted in well-established interventions for symptomatic alleviation (Marnach et al., 2022). Antibiotics, vaginal douching, antibacterial detergents, pH modulators, and hormones are commonly used to treat vaginitis, but they often provide short-term relief, leading to a high recurrence rate (Mitchell, 2024). This clinical challenge has prompted a strategic shift in research focus toward innovative interventions specifically designed to modulate both the compositional architecture and functional dynamics of the VMB, with the ultimate objective of achieving durable therapeutic outcomes (Figure 3). These emerging modalities collectively represent a transformative advancement in vaginal healthcare, offering distinct yet complementary mechanisms for precise and sustained microbiome modulation, thereby addressing the critical limitations of conventional treatment strategies (Mohankumar et al., 2022).

### 3.1 Prebiotics

Prebiotics, such as inulin, fructooligosaccharides, pectin, and sugar alcohols, serve as nutritional substrates that selectively stimulate the growth and activity of beneficial microbes within the host. The prebiotic lactulose promotes the growth of *Lactobacillus* while inhibiting the growth of BV-related species or *C. albicans*. Its use is instrumental in preserving vaginal homeostasis, preventing dysbiosis, and consequently, reducing the susceptibility to infection (Collins et al., 2018). In several clinical trials, lactoferrin has demonstrated positive effects in clinical trials involving women with reproductive tract ecological disorders and those with bacterial and fungal infections that pose a risk to preterm labor (Otsuki and Imai, 2017; Russo et al., 2018, 2019a,b). Pectin is a complex carbohydrate with a gel-like consistency, functions as an effective delivery system for vaginal probiotics or antibiotics. Its rheological properties make it an ideal carrier, enhancing the retention and distribution of therapeutic agents within the vaginal environment (Vigani et al., 2019; Conte et al., 2024). Studies have indicated that the inclusion of pectin in the diet can lead to a significant increase in *Lactobacillus* populations in the vagina, as evidenced in sows, suggesting that pectin may offer benefits for maintaining female vaginal health (He et al., 2024).

### 3.2 Postbiotics

Postbiotics are composed of non-viable microbes or their derived components, offer health benefits to the host. They exert their effects through the regulation of various bioactive substances, similar to the mechanisms of probiotics (Salminen et al., 2021). A recent study showed that a postbiotic gel effectively reduced symptoms of BV and in restoring to the balance of the vaginal bacteria community (Shen et al., 2023). Furthermore, exopolysaccharides produced by *Lactobacillus* can promote beneficial biofilm development and hinder the formation of biofilms by harmful pathogens (Giordani et al., 2023). Extracellular vesicles released by *Lactobacillus* have been found to enhance adherence to HeLa cells and serve as a barrier against pathogen adherence (Croatti et al., 2022). Consequently, these postbiotic components, such as exopolysaccharides and extracellular vesicles, may offer significant benefits in the prevention and treatment of vaginal infections.

### 3.3 Probiotics

The combination of probiotics with antibiotics is more effective in treating BV and reducing the risk of recurrence compared to antibiotics alone (Afifirad et al., 2022). This synergistic approach not only addresses the immediate infection but also helps to reestablish a balanced vaginal microbiome, which is crucial for long-term health. Similarly, the integration of probiotics with antifungal therapies has proven to be advantageous in managing both simple and complex cases of VVC, leading to decreased recurrence rates and an enhanced vaginal microenvironment (Martinez et al., 2009; Zeng et al., 2023). Common probiotic strains utilized in clinical settings include *L. reuteri*, *L. crispatus*, *L. rhamnosus*, *L. acidophilus*, *L. delbrüclister*, and *L. gasseri* (Ling et al., 2013; Tomusiak et al., 2015; Ho et al., 2016; Dausset et al., 2018; Cohen et al., 2020; Sgibnev and Kremleva, 2020). These strains are often administered in the form of oral tablets or capsules, as well as vaginal capsules or suppositories, to treat conditions such as BV, VVC, and AV. The dosage typically ranges from 2.5 × 10^6^ to 1 × 10^10^ colony-forming units (CFU), and research has demonstrated the efficacy of both administration routes (Gupta et al., 2024). However, the exact efficacy may be influenced by various factors, including the type of probiotics, dosage size, frequency of use, and the individual physiological characteristics of the patient. Both oral and vaginal administration routes can effectively increase the number of *Lactobacillus* in the vagina and correspondingly reduce the proliferation of harmful pathogens. The impact of oral probiotics on the composition of VMB may stem from the migration of probiotics through the gut-vaginal axis or indirectly promote vaginal health by modulating the overall immune system function (Han and Ren, 2021). In clinical practice, vaginal probiotic capsules or suppositories are often prescribed sequentially with antibiotics to patients. Table 1 provides an overview of specific studies and their findings. Several clinically validated probiotic formulations have received pharmaceutical-grade certification, including Dingjunsheng^®^, EcoVag^®^, Gynoflor^®^, and in-Vag^®^.

**TABLE 1:** Studies on preparations of probiotics used to restore the vaginal microecosystem.

Pharmaceutical form or product	Probiotic components	Specification	Drug	Country	Clinical target	Instructions for use	Characteristics and number of participants	Conclusions	References
Dingjunsheng^®^ vaginal capsule	*L.* .*delbrueckii* DM8909	5 capsules/pack	Yes	China	BV	Once daily at bedtime for 10 days	123 metronidazole, 144 probiotics	Probiotics significantly reduced the recurrence rate	Zhang et al., 2025
EcoVag^®^ vaginal capsule	*L. rhamnosus* DSM 14870, *L. gasseri* DSM 14869	10 capsules/box	Yes	Denmark	BV/RVVC	Antibiotic treatment followed by probiotics	40 Scandinavian BV or VVC-diagnosed women	Combined use effectively provides long-term cures for BV and RVVC	Pendharkar et al., 2015
					Preterm PROM	Probiotics for 10 days in combination with antibiotic prophylaxis	49 cases probiotics and antibiotic, 57 cases antibiotic	Probiotics prolonged the latency period and improved the perinatal outcome	Daskalakis and Karambelas, 2017
Gynoflor^®^	*L. acidophilus* KS400	6, 12, or 36 tablets/box	Yes	Switzerland	Atrophic vaginitis	One intravaginal tablet daily for 28 days	16 postmenopausal breast cancer	The combination of *Lactobacillus* and estriol rapidly and sustainably improved the vaginal microbiome in women with painful BC	Donders et al., 2015
NORMOGIN™ vaginal tablet	*L.* .*rhamnosus* BMX 54	6 tablet/bottle	Yes	Italy	Restores a balanced vaginal ecosystem	Metronidazole and vaginal *Lactobacillus* for 3 months Fluconazole and vaginal *Lactobacillus* for 6 months	117 HPV + BV or VVC-women	Supplementation with probiotics helps clear HPV	Palma et al., 2018
inVag^®^ vaginal capsule	*L. fermentum* 57A, *L. plantarum* 57B, *L. gasseri* 57C	7 capsules/pack	Yes	Poland	VM modulation	One capsule daily for 7 days	160 Vaginal disorders	Restores a healthy VMB	Tomusiak et al., 2015
Lactin-V	*L. crispatus* CTV-05	–	Yes	America	BV	Metronidazole gel treatment followed by Lactin-V for 11 weeks	152 to the Lactin-V group, 76 to the placebo group	Probiotic supplementation lowers BV recurrence rates	Cohen et al., 2020
prOVag^®^ oral capsule	*L. fermentum* 57A, *L. plantarum* 57B, *L. gasseri* 57C	10 or 20 capsules/pack	No	Poland	BV/AV	Antibiotics and oral probiotic capsules for 10 days	118 BV/AV	Oral probiotics lengthen remission in patients with recurrent BV/AV	Heczko et al., 2015
Ecocillin^®^ oral capsule	*L. plantarum* P17630	15 capsules/pack	No	Italy	RVVC	3 treatment cycles (15 days/cycle) separated by 15-day wash-out intervals	93 RVVC	Prevents episodes of VVC	Vladareanu et al., 2018
Gynophilus^®^ LP vaginal capsule	*L.rhamnosus* Lcr35	2,6,12 capsule/pack	No	France	VM improvement	Take every 3, 4, or 5 days or daily for 21 days	33 healthy women	Protects a healthy vaginal microbiome	Dausset et al., 2018
					TV with BV infection	Gynophilus, in combination with metronidazole, twice daily for 7 days	49 TV + BV	Probiotic increased TV and BV cure, decreased vaginal inflammation	Sgibnev and Kremleva, 2020
Lactagyn^®^vaginal capsule	*L. acidophilus*, *L. rhamnosus*, *S. thermophilus*, *L*. *delbrueckii* subsp. *bulgaricus*	10 capsules/pack	No	Bulgaria	RVVC	Start intravaginal capsules on the 5th day after antibiotic therapy	201 RVVC	Probiotic supplementation improves treatment efficacy and prevents relapse	Kovachev and Vatcheva-Dobrevska, 2015
UREX	*L. rhamnosus* GR-1, *L. reuteri* RC*-*14	–	No	Denmark	GBS	Immediately after diagnosis, take the capsule orally until delivery	49 in the probiotic	Probiotics reduce the vaginal and rectal GBS colonization rate in pregnant women	Ho et al., 2016
					BV	Receive metronidazole treatment for 7 days and take oral probiotics for 30 days	49 in the probiotic	Probiotics and antibiotic in the eradication of BV in black African women	Anukam et al., 2006
					BV	Receive metronidazole treatment for 7 days and take oral probiotics for 30 days	60 in the probiotic	Combined oral probiotics and metronidazole did not improve BV cure rate vs. metronidazole alone in Chinese patients.	Zhang et al., 2021

AV, aerobic vaginitis; BV, bacterial vaginitis, GBS, group B *Streptococcus*; HPV, human papilloma virus; OTC, over-the counter; PROM, premature rupture of membranes; RVVC, recurrent vulvovaginal candidiasis; VMB, vaginal microbiota; TV, trichomonas vaginitis; VVC, vulvovaginal candidiasis.

### 3.4 Synbiotics

Synbiotics, which are synergistic formulations combining probiotics and prebiotics, leverage the prebiotics to enhance the survival and functionality of the probiotics. In a prospective, randomized, double-blind, placebo-controlled pilot study, VagiBIOM vaginal suppositories demonstrated significant improvements in the overall vaginal health index and provided substantial relief from vaginal itching in patients with BV after a 4-week intervention period. Microbiome analysis revealed that these improvements were largely attributable to an enhancement in the diversity of vaginal *Lactobacillus* (Vivekanandan et al., 2024).

### 3.5 Vaginal microbiota transplantation (VMT)

Vaginal microbiota transplantation involves the transfer of a healthy VMB from a donor to a recipient, to restore balance to the recipient’s VMB and improve overall health. Lev-Sagie et al. (2019) reported that VMT was used to treat patients with BV, and it was found to improve the appearance of vaginal fluid and to reestablish a *Lactobacillus*-dominated VMB after a follow-up period of 5–21 months. Remarkably, there was a case where a woman with a VMB consisting of 90% *Gardnerella* spp. underwent VMT without receiving antibiotic treatment. This direct approach resulted in a significant shift in her microbiota composition to 81.2% *L. crispatus*, which remained stable for 1.5 years. Following the VMT, she conceived 5 months later and delivered a healthy infant (Wrønding et al., 2023).

## 4 Live biotherapeutics: recent advances and development strategies

### 4.1 Overview of live biotherapeutic products

Products containing live microorganisms are regulated and utilized in distinct categories based on their intended use, level of clinical validation, and legal status. Dietary probiotics are generally classified as foods or supplements, intended to support general wellbeing and regulated primarily for safety without requiring proven therapeutic efficacy. Pharmaceutical probiotics are approved as drugs, supported by clinical trials for the prevention or treatment of specific diseases, and are often available by prescription. At the most advanced end of this spectrum, LBPs represent a novel class of biologic drugs that undergo comprehensive pharmaceutical development and rigorous clinical validation to precisely prevent, treat, or manage defined medical conditions through targeted modulation of the host microbiome.

Live biotherapeutic products are defined by the U.S. Food and Drug Administration (FDA) as biological products that contain live organisms and are intended for the prevention, treatment, or cure of a disease, excluding vaccines. LBPs are prescription pharmaceuticals comprising viable microorganisms-including bacteria, yeast, or carefully selected strain combinations-that have undergone full pharmaceutical development for targeted disease intervention (FDA, 2016; Pribyl et al., 2025). Importantly, not all probiotic formulations qualify as LBPs, as regulatory classification depends on intended use, clinical claims, and jurisdictional frameworks (Cordaillat-Simmons et al., 2020; Tseng et al., 2025). In the context of vaginal health, these advanced biologics, which include certain probiotic formulations (particularly *Lactobacillus*-based preparations) and VMT therapies, function through direct microbial ecosystem engineering.

As of now, China has not yet promulgated a standalone regulatory framework specifically governing LBPs. Regulation of these products currently relies on the existing drug regulatory system, with adaptations made to address the unique characteristics of biologics. Additionally, reference is made to the FDA’s guidance document for industry stakeholders titled Early Clinical Trials with Live Biotherapeutic Products: Chemistry, Manufacturing, and Control Information.

### 4.2 Recent advances

As shown in Table 1, probiotic preparations successfully marketed for vaginal use encompass various types, including single-strain and multi-strain formulations. Since these products were approved early, they may not yet be classified as LBPs according to FDA definitions. Beyond these commercially available products, recent years have witnessed remarkable advancements in novel LBPs for treating gynecological infectious diseases, particularly demonstrating exceptional therapeutic potential for vaginal infection, like Lactin-V (*L*. *crispatus* CTV-05) (Armstrong et al., 2022; Hemmerling et al., 2025).

Furthermore, according to the clinical trial registration information from China’s National Medical Products Administration (NMPA), the Phase III clinical trial (CTR20210714) evaluating the combination therapy of vaginal *Lactobacillus* live capsule (a single-strain formulation) with metronidazole was completed in November 2023. This achievement has established a solid clinical foundation for the application of single-strain formulations in the treatment of gynecological infectious diseases. The development of multi-strain formulations has also entered a critical phase. In June 2024, a Phase III study (CTR20241592) on a vaginal *Lactobacillus* dual-strain live capsule was initiated, while a Phase IIb trial (CTR20240668) investigating sequential therapy with metronidazole followed by a vaginal *Lactobacillus* quadruple-strain live capsule commenced smoothly in May 2024. These studies focus on exploring the mechanisms and clinical efficacy of multi-strain synergistic therapy, offering the potential for more comprehensive treatment options for gynecological infections.

In the field of microbiome modulation, VMT (NCT04517487) has emerged as an innovative therapeutic strategy. Restoring a *Lactobacillus-*dominant vaginal microecological balance provides a new treatment option for patients with recurrent BV. However, this technology still faces critical scientific challenges, including standardization of microbiota preparation, optimization of transplantation protocols, and long-term safety assessments, necessitating further in-depth research to advance its clinical application.

From single-strain to multi-strain formulations and further to microbiota transplantation, LBPs have preliminarily established a multi-tiered therapeutic system. This framework offers novel approaches and methodologies for the precision treatment of gynecological microecology-related diseases, holding significant clinical value and broad application prospects.

### 4.3 Development strategies

In terms of strain selection, current clinical research primarily focuses on vaginal probiotic strains such as *L*. *crispatus*, *L*. *gasseri*, and *L*. j*ensenii*. Among these, *L*. *crispatus* has garnered significant attention due to its status as the dominant VMB in healthy women. Its strong colonization capacity, stable acid-producing properties, and robust clinical evidence (such as its proven effectiveness in significantly reducing the recurrence rate of BV) make it the preferred choice.

When developing dosage forms, it is necessary to comprehensively consider factors such as the stability of the drug, patient compliance, and the drug’s release characteristics. Capsules are a common dosage form. Their advantage lies in their ability to act directly on the local area of the vagina, avoiding the potential gastrointestinal effects that oral medications may encounter. In addition, the capsule form is convenient for patients to use and can, to a certain extent, protect live bacteria from the influence of the external environment, ensuring the drug’s effectiveness (Lima et al., 2025). However, during the development process, issues such as the long-term stability of live bacteria and encapsulation technology still need to be addressed to ensure the activity of the drug during storage and use.

The development of LBPs typically commences with bioinformatics analyses to identify promising microbial strains. Microbe-seq technology facilitates high-throughput, culture-independent analysis of complex microbial communities at the single-cell level (Zheng et al., 2022). Droplet microfluidic culture enables the isolation and cultivation of microbial strains within vast numbers of picoliter-sized droplets (Meng et al., 2022), with subsequent strain identification possible through methods such as phenotype microarrays, 16S rRNA sequencing, or MALDI-TOF MS (Carvalho et al., 2022). For preliminary functional screening and to assess the safety and efficacy of LBPs intended for vaginal health, a vaginal chip composed of primary vaginal epithelial cells and stromal fibroblasts is employed (Mahajan et al., 2022). Subsequent investigations into safety and efficacy are conducted at bacterial, cellular, and animal levels. Given the sensitivity of probiotics to external environmental factors, technological advancements in processing are vital for enhancing delivery efficiency. Nanotechnology, particularly electrospinning, has emerged as a potential method for the one-step encapsulation of active lactic acid bacteria, offering an increase in the viability of vaginal strains compared to traditional freeze-dried powders (Silva et al., 2021).

**FIGURE 4:**
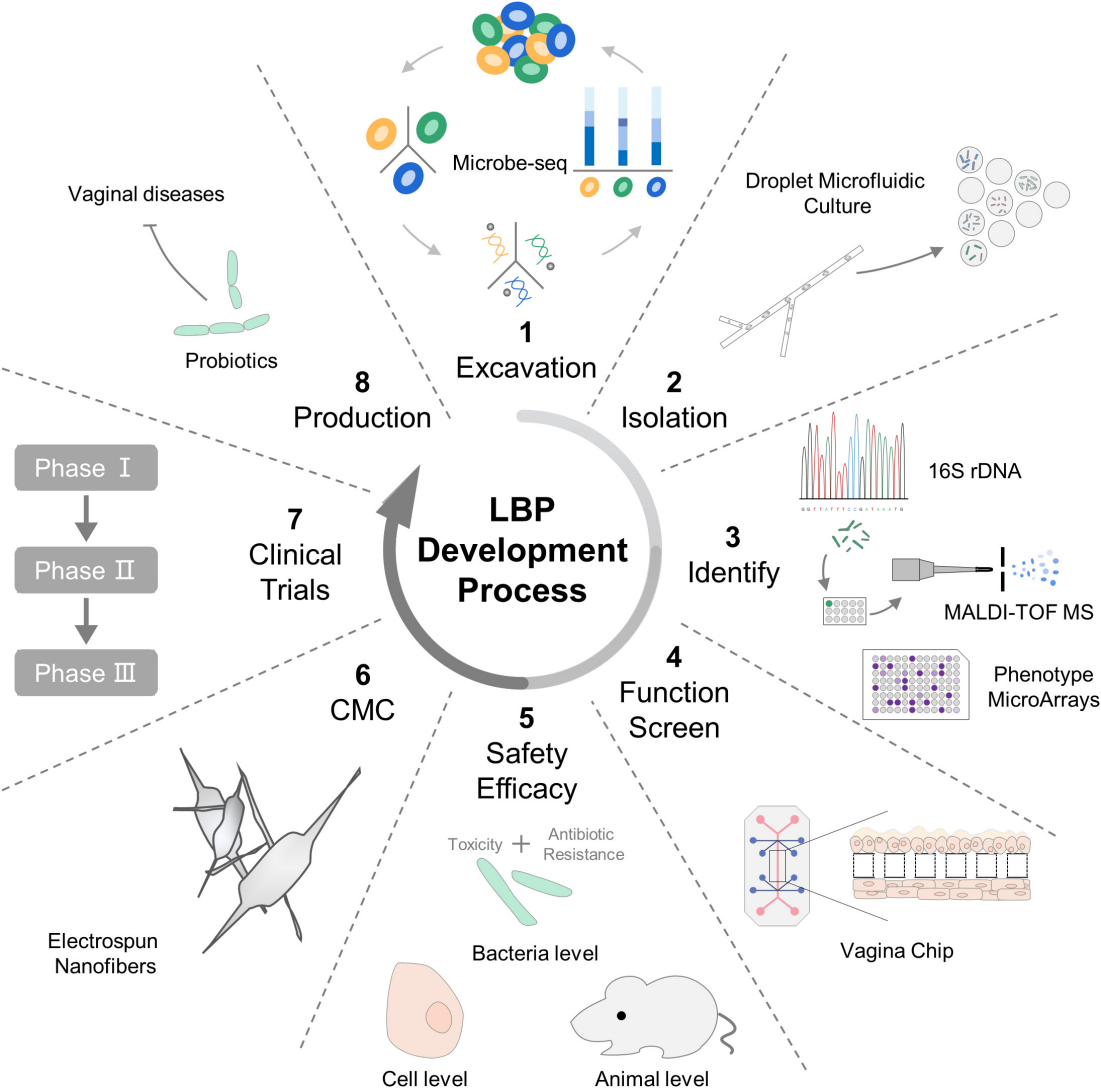
Processes and advances in the development of live biotherapeutic products (LBPs). LBPs research and development usually begins with bioinformatics analyses to identify candidate strains. Microbe-seq is used for high-throughput culture-free analysis to investigate genomic blueprints of complex microbial communities with single-microbe resolution. Next, droplet microfluidic culture enables the isolation and culture of strains in millions of picoliter droplets. Afterward, phenotypic microarrays, 16S rDNA sequencing, or MALDI-TOF MS are used to identify the isolated strains. The vagina chip is lined by primary vaginal epithelium interfaced with underlying stromal fibroblasts, enabling preliminary function screening for vaginal probiotics and safety and efficacy evaluations of LBPs. The safety and efficacy data are then further investigated in the bacterial, cell, and animal models. Clinical research is conducted when product manufacturing control is completed. The use of electrospun nanofibers is an effective technology that enables the high survival rate and long-term viability of probiotics. Clinically proven, effective, and safe LBPs are used to treat vaginal infections or illnesses, as well as to maintain VMB homeostasis. CMC: chemical manufacturing control, MALDI-TOF MS: matrix-assisted laser desorption/ionization time-of-flight mass spectrometry.

The aforementioned studies have substantially advanced the field of human microecology by deepening our insight into novel mechanisms, facilitating the screening and cultivation of microbial strains, and validating their functional roles. To ensure the robustness and generalizability of these findings, it is crucial to conduct large-scale clinical studies with extensive sample sizes obtained from a variety of institutions. Such research endeavors will be instrumental in the development of tailored and precision-based vaginal microecological products, ultimately contributing to the improvement of women’s health (Figure 4).

### 4.4 Challenges and limitations

Despite the promising prospects of LBPs, their translation from laboratory research to clinical application still encounters multidimensional challenges spanning from fundamental mechanistic studies to final market access. This section aims to systematically outline the major scientific, clinical, and regulatory bottlenecks currently faced by LBPs, with a particular focus on interventions targeting the VMB.

#### 4.4.1 The complexity of safety and efficacy assessment

The clinical translation of LBPs is first and foremost constrained by the inherent complexity of conducting comprehensive assessments of their safety and efficacy. Regarding safety, a primary challenge lies in the efficiency and persistence of exogenous live bacteria at the target site. Multiple clinical studies indicate that even after antibiotic pre-treatment, the colonization rate of LBPs in sites like the vagina remains suboptimal (∼49%), and individuals who fail to achieve colonization exhibit a recurrence risk comparable to the placebo group, severely limiting their long-term efficacy (Potloane et al., 2025). Secondly, there is a current lack of a systematic safety assessment framework tailored to specific anatomical sites such as the female reproductive tract. Existing standards inadequately incorporate critical indicators like vaginal irritation scores and cervical cytological changes, while also lacking reliable *in vitro* or *in vivo* models to simulate and evaluate the risk of bacterial translocation (Rouanet et al., 2020). Most critically, for special populations such as pregnant women, immunocompromised individuals, and neonates, the application of LBPs carries a theoretically significant safety risk. Given the compromised physiological barriers in these groups, introducing live bacteria could lead to bacteremia, disrupt maternal-fetal homeostasis, or trigger horizontal gene transfer. Therefore, based on prudent extrapolation from pathophysiological mechanisms, classifying such populations as relative or absolute contraindications or LBPs is the prevailing view in the field pending prospective safety data. In terms of efficacy, the challenge primarily resides in establishing a robust efficacy evaluation system. Although the FDA has recommended composite endpoints combining symptom resolution with microbial community stability, a consensus on the definition and quantification of a “protective microbiome” remains elusive (Valeriano et al., 2024), creating substantial uncertainty for clinical trial design and result interpretation. Furthermore, existing clinical trials universally suffer from insufficient population diversity, with study cohorts often limited to specific geographic regions and physiological states, thereby restricting the generalizability of the findings.

#### 4.4.2 Lessons from other therapeutic areas

The developmental trajectory of LBPs has not been without setbacks, and clinical trial outcomes in other indications offer valuable lessons and warnings for vaginal LBP research. On the one hand, several programs have been terminated due to insufficient efficacy. For instance, Forte Biosciences’ *Roseomonas mucosa*-based FB-401 failed to meet the primary endpoint in a Phase II trial for atopic dermatitis (Fronte Biosciences Inc, 2021), and Synlogic’s engineered *Escherichia coli* Nissle 1917 strain, SYNB1934, was discontinued in a Phase III trial for phenylketonuria due to poor efficacy data (Synlogic, 2024). These cases underscore that the efficacy of LBPs is not a foregone conclusion; their success hinges on multiple variables, including strain selection, dose optimization, and patient stratification. Conversely, some programs have achieved success through strategic refinement, as exemplified by Seres Therapeutics’ SER-109, which, after failing a Phase II trial, ultimately succeeded in a Phase III study following a revised protocol and received FDA Breakthrough Therapy designation (McGovern et al., 2021; Kraft et al., 2024). Together, these contrasting experiences demonstrate that the clinical success of LBPs is a synergistic outcome of multiple factors, encompassing microbial colonization capacity, rational dosing strategies, and a profound understanding of inter-individual heterogeneity in the host microbiome.

#### 4.4.3 Unique dilemmas and market disarray in the vaginal microbiome field

Compared to other domains, the development of vaginal LBPs presents some distinctive dilemmas. Although numerous candidate products have entered late-stage clinical development, to date, no publicly reported program has been formally terminated due to colonization failure. This phenomenon may stem from sponsors’ efforts to mitigate early-stage safety risks, but it could also mask latent, yet-to-be-identified efficacy bottlenecks. Concurrently, the market is saturated with probiotic products claiming to restore VMB balance. However, these products largely lack support from rigorously designed randomized, double-blind, placebo-controlled trials, reflecting a severe deficiency in clinical validation and a dearth of scientific evidence within the field. This juxtaposition of scientific fervor and market disarray further highlights the urgency of establishing a standardized clinical evaluation framework.

While it is true that current clinical data have not reported serious adverse events associated with the use of probiotic products in special populations such as pregnant women, this provides initial confidence for future research (Yang et al., 2020b; Petricevic et al., 2023; Binda et al., 2025). However, the journey from an absence of evidence for harm to conclusive evidence of benefit and safety remains long. Data concerning strain specificity, dose-response effects, and long-term impacts remain scarce. Therefore, we must not be complacent about the current safety record but should instead commit to designing and executing more rigorous clinical trials to fill these critical knowledge gaps.

#### 4.4.4 Regulatory pathway uncertainty

Finally, the commercialization of LBPs faces the challenge of regulatory pathway ambiguity. As LBPs possess characteristics of both conventional drugs and biologics, their regulatory framework must satisfy dual requirements, objectively increasing development complexity and cost (Rouanet et al., 2020). To date, no vaginal live biotherapeutic has been approved in the European Union, indicating that global regulatory agencies still require time to establish unified and clear approval standards and guidelines.

## 5 Conclusion

In conclusion, the VMB, particularly its enrichment with *Lactobacillus* species, is undeniably fundamental to women’s health. While traditional therapies for dysbiosis face persistent challenges, including high recurrence rates and antibiotic resistance, the advent of LBPs offers a promising paradigm shift. However, translating this potential into clinical reality requires overcoming significant hurdles. Our analysis reveals that substantial gaps remain in our understanding of VMB dynamics, including the precise definition of a healthy state, the complex interplay between the host and its microbial inhabitants, and the long-term safety and efficacy of LBPs, especially in vulnerable populations. Therefore, moving forward, a concerted effort is needed. This includes conducting large-scale, rigorously designed clinical trials with robust endpoints, establishing standardized regulatory pathways, and fostering interdisciplinary collaboration. Ultimately, bridging these gaps is not just a scientific pursuit but a critical step toward developing effective and safe interventions that can truly transform women’s reproductive health.
